# Pulmonary effects of repeated six-hour normoxic and hyperoxic dives

**DOI:** 10.1371/journal.pone.0202892

**Published:** 2018-09-07

**Authors:** Barbara E. Shykoff, John P. Florian

**Affiliations:** Navy Experimental Diving Unit, Panama City, Florida, United States of America; Emory University School of Medicine, UNITED STATES

## Abstract

This study examines differential effects of immersion, elevated oxygen partial pressure, and exercise on pulmonary function after series of five daily six-hour dives at 130 kPa (1.3 ATA), with 18 hours between dives. Five cohorts of 10 to 14 divers participated. The exposure phases were resting while breathing O_2_ or air in the water (“wetO_2_”, “wetAir”) or O_2_ in the hyperbaric chamber (“dryO_2_”), and exercise in the water while breathing O_2_ or air (“wetO_2_X”, “wetAirX”). Respiratory symptoms were recorded during and after each dive, and pulmonary function (forced flow-volume) was measured twice at baseline before diving, after each dive both immediately and on the following morning, and three days post diving (“Day+3”). The incidences of symptoms and of flow volume changes from baseline greater than normal limits (“ΔFV”) were assessed, as were mean ΔFV. The parameters examined were forced vital capacity (FVC), forced expired volume in 1 second (FEV_1_), and forced expired flow from 25% to 75% volume expired (FEF_25–75_). The phases ranked from greatest to least fraction of diver-days with symptoms were wetO_2_X (56%) > dryO_2_ (42%) > wetO_2_ (13%) > [wetAir (2%) or wetAirX (1%)] (p<0.05). FEV_1_ and FEF_25–75_ were depressed in the morning following wetO_2_ and wetO_2_X and on Day+3 after and wetO_2_X, but increased immediately following each wetAirX dive. O_2_ exposures caused symptoms and ΔFV suggestive of pulmonary oxygen toxicity,exacerbated by exercise. Indices of small airway function showed late (17-hour) post-O_2_ exposure deficits, but, particularly with exercise, improvement was evident early after exposure with or without O_2_. FEF_25–75_ and FEV_1_ remained depressed on Day+3 after wetO_2_ and wetO_2_X.

## Introduction

Pulmonary oxygen toxicity is a known hazard of long exposures to oxygen partial pressure (PO_2_) greater than about 50 kPa (0.5 atm) [1]. In previous work, single long duration (four- to eight-hour) dives with PO_2_ = 130 to 140 kPa (1.3 to 1.4 atm) provoked mild pulmonary oxygen toxicity in some individuals whether divers on the bottom were resting [2] or exercising intermittently on cycle ergometers for four hours [3]. When four-hour dives were repeated daily for five days, pulmonary symptoms and changes in pulmonary function were minimal for divers who rested on the bottom. However, when the divers exercised on the bottom reports of symptoms and measured signs suggestive of pulmonary oxygen toxicity increased across the dive week, and some divers complained of excessive fatigue and exercise intolerance [3]. When six-hour resting dives were repeated, divers reported extreme fatigue and some cumulative pulmonary effects were evident [2].

The further investigation of the pulmonary effects of six hour dives with PO_2_ of 130 kPa with divers resting is a continuation of the previous work. Measurement after six hour dives while divers exercised is an extension of it. Of interest are which effects might be due to hyperoxia, which to immersion, and which to immersed hyperoxia; normobaric hyperoxia without immersion is known to cause some direct oxidative damage to tissues and or surfactant [4,5]. Head out water immersion alters hemodynamic and pulmonary function, and the effects of fluid shifts from long immersions persist long after emergence from the water. Divers who are totally immersed and breathing from a demand regulator at the mouth have pulmonary static loading equivalent to that of head-out water immersion in the matching posture. Water immersion or submersion causes blood volume to move from the legs and splanchnic bed into the thorax [6,7]. Resting end-expiratory lung volume decreases and pulmonary congestion follows [8]. The increase in pulmonary blood volume is great enough that it decreases vital capacity [9]. Further, airway flow resistance increases because of reduced lung volume [10], increasing the magnitude of pressure swings needed to generate inspiratory flow. The increased output of the left ventricle limits the increase in pulmonary congestion but does not eliminate it [11]. Thus, a diver breathing in a head-up position underwater with a demand regulator in his or her mouth 1) must generate increased pressure swings to overcome the static load and the increased airway resistance; and 2) experiences pulmonary congestion because of the translocation of blood into the chest. Exercise in the water exacerbates both effects [12]. These large intrathoracic pressure swings in the presence of pulmonary congestion, repeated for hours and then for days, might be expected to cause post-exposure pulmonary symptoms and changes in pulmonary function even without hyperoxia. This study compared those effects of breathing underwater with those of breathing hyperbaric oxygen to answer questions about pulmonary effects of repeated, long-duration dives.

To partition the effects of hyperoxia, immersion, and exercise, five phases of five, six-hour dives repeated daily (“dive weeks”) were conducted: oxygen breathing underwater by divers at rest (“wetO_2_”); air breathing underwater by resting divers (“wetAir”); oxygen breathing in the hyperbaric chamber at the same partial pressure as that in the water, also by resting subjects (“dryO_2_”); oxygen breathing underwater by divers who exercised: (“wetO_2_X”); and air breathing underwater by divers who exercised (“wetAirX”). The pulmonary data–symptoms that may indicate pulmonary oxygen toxicity, and specific forced flow-volume indices (forced vital capacity [FVC], forced expired volume in 1 second [FEV_1_], and forced expired flow from 25% to 75% volume expired [FEF_25–75_] from those phases are presented here. Differential effects of mild hyperoxia, submersion and negative pressure breathing, and exercise are discussed.

The physiology of special environments–e.g., hyperoxia, immersion, altitude–has been studied mostly when various government and military programs needed specific information, and funding has been sparse for any studies when this was not the case. Aviation and oxygen diving physiology were studied intensively during the Second World War and immediately after. Immersion studies were funded extensively as surrogate microgravity exposures as the space program began and later, as long sojourns in space were contemplated. Diving research over the last decade or so, including this work, has been driven by the desire to spend longer underwater. Available references reflect these cycles of funding.

## Methods

### Ethics, consent, and permissions

All studies were approved by the Institutional Review Board at Navy Experimental Diving Unit. Each subject gave written informed consent before participating, and all procedures conformed to the Declaration of Helsinki.

### Subjects

Healthy U.S. Navy divers dove for six hours per day on five consecutive days, with 18-hour surface intervals (SI). Before and after each day’s diving, forced flow-volume loops (forced vital capacity maneuvers) were recorded using a spirometer-based pulmonary function machine (CPL, nSpire Medical, Longmount CO). The loops were examined for quality and consistency of values, but only FVC, FEV_1_, and FEF_25-75_were compared statistically and are presented. The times of measurement for each individual were consistent. Morning measurements were made from 5:30 AM to 8:30 AM and afternoon measurements from about 2 PM to about 5 PM. Forced vital capacity maneuvers were also conducted within the week before diving, on the morning following the fifth dive, and three days after the fifth dive. The average of parameters from three flow-volume loops reproducible according to American Thoracic Society standards [13] was used at each time point, and was compared to a baseline average of six reproducible values, three from the week before diving and three from the morning before the first dive. In-water (“wet”) dives were conducted in a 4.6 m (15 foot) deep, fresh water pool, kept comfortably warm (31.7 ± 1.7°C [89 ± 3°F] rest, 30.6±1.7°C [87±3°F] exercise). At those temperatures the divers needed no thermal protection, and wore just shorts and T-shirts, as they did in the lab before submersion and after changing into dry clothes after diving. Divers with lung centroid at a depth of 3.4 to 4.0 m (11 to 13 feet) breathed surface-supplied, humidified gas, open circuit from full face masks with self-contained demand regulators (AGA Divator mask, Interspiro, Cliffwood Beach NJ).

Up to four divers participated during any dive week. They entered the water individually at intervals of about an hour. After three hours of immersion they surfaced to stand in shoulder-deep water and breathe room air during a 10-minute lunch break. The breathing gas underwater was either air (“air dives”) or 100% oxygen (“O_2_ dives”), for a PO_2_ of approximately 30 or 130 kPa (0.3 or 1.3 atm), respectively. Divers either rested in chairs on the bottom (“resting dives”) or exercised prone but about 30° head-up on in-water cycle ergometers (“exercise dives”). During exercise dives, divers alternated 30 minutes of seated rest with 30 minutes of exercise, and ergometer brake power was adjusted to generate heart rates of 90 to 110 beats/min. The load was an attempt to mimic the workload of a hypothetical average dive without current.

Five different sets of dive conditions were investigated, four in the water and one in a dry hyperbaric chamber. Different divers participated in the five phases. Dry hyperbaric exposures (“dry dives”) were conducted in a large hyperbaric chamber that was compressed with air to 130 kPa. Seated divers breathed humidified oxygen from hoods (Amron 8891, Amron International, Vista, CA). Divers locked into the chamber individually at approximately one hour intervals. They removed the hoods after three hours exposure to breathe chamber air and eat and drink for no more than 10 minutes before resuming oxygen breathing.

Specific symptoms—inspiratory burning, cough, chest tightness or dyspnea—were considered to indicate pulmonary oxygen toxicity. Subjects were queried about symptoms, which were scaled as “none”, “mild”, “moderate”, “moderately severe”, or “severe”, whenever pulmonary function was measured and hourly during the dives. A diver who reported any symptom from that list was considered to have possible pulmonary oxygen toxicity on that day (S1 Table, S2 Table). When the fraction of person-days with symptoms was calculated, a full diver-day was allocated.

Changes in flow-volume parameters that fell outside the normal non-diving 95% confidence limits as previously measured at the Navy Experimental Diving Unit [2] also were considered to be evidence of pulmonary oxygen toxicity. Specifically, decreases from baseline in FVC greater than 7.7%, in FEV_1_ greater than 8.4%, or in FEF_25–75_ greater than 16.8% were considered to be deficits, collectively termed “ΔFV”. Incidences of symptoms and of ΔFV were compared across dive conditions using Fisher’s Exact Test.

To account for differences across dive phases, fractional changes from baseline in pulmonary function indices were compared rather than the raw values. The average fractional changes from baseline in flow-volume parameters FVC, FEV_1_, the ratio of FEV_1_/FVC, and FEF_25–75_ were assessed across dive weeks.

For the in-water data, the initial statistical analysis was an analysis of variance (ANOVA) with two between-subjects factors, gas (oxygen or air delivered) and rest or exercise, with repeated measures on the within-subject factors dive day (Dives 1 to 5) and time of measurement (immediately after a dive [“PM”] or the following morning [“AM”]). Differences were considered to be significant if α = 0.05 after Greenhouse-Geisser (G-G) correction for non-sphericity as appropriate. If a significant interaction of gas was found with rest and exercise or with time of day, repeated measures ANOVAs were repeated on each of the conditions, that is, one ANOVA for rest and one for exercise, or one for AM and one for PM. Further interactions including gas were similarly investigated, then time of measurement, with the final drilling-down a Şidak-corrected pairwise comparison of dive days and comparison of dive days to baseline.

The resting oxygen dives in the hyperbaric chamber (DryO_2_) were compared to the resting in-water oxygen dives (WetO_2_) with a one factor (wet or dry) repeated measures ANOVA. Measurements within the condition were made using repeated measures ANOVA with no factors. Follow-up measurements were compared to baseline using t-tests.

## Results

Numbers of divers and their characteristics are presented by type of dive in Table 1.

All participants were healthy and had met physical examination criteria for diving. Nevertheless, a number had baseline FEV_1_/FVC ratios lower than 75%: one diver for wet resting O_2_, two for wet resting air, one for dry resting O_2_, two for wet exercise O_2_, and five for wet exercise air. Divers were included in the study regardless of baseline FEV_1_/FVC ratio. All analysis was of comparison to baseline, and population normative values were not considered.

**Table 1:** Subject characteristics.

Dive week type	Number	Age (Years)	Height (cm)	Weight (kg)
Wet resting O_2_	10	33 (28–44)	179 (175–185)	86 (75–100)
Wet resting air	10	36 (19–44)	178 (170–191)	85 (73–98)
Dry resting O_2_	12	28 (20–36)	180 (173–193)	86 (71–102)
Wet exercise O_2_	10 (12)	25 (20–44)	178 (168–185)	82 (68–105)
Wet exercise air	14	32 (23–44)	182 (173–193)	84 (71–107)

Values listed are medians, with the minima and maxima in parentheses. Parentheses in the “Number” column indicate the number of divers for whom data are included for only part of the week. Two divers withdrew from the wet exercise O_2_ weeks with symptoms of pulmonary oxygen toxicity.

### Occurrence of pulmonary signs and symptoms

The fractions of divers who reported symptoms or who had measurable changes in flow-volume (ΔFV) parameters are reported in Table 2 for the five phases. Divers frequently reported symptoms without measurable flow-volume deficits (“signs”), and occasionally vice versa; symptoms, signs, and both (i.e., any pulmonary oxygen toxicity) are tabulated individually. The incidences include those reported during a dive, those evident immediately post-dive and those first measured or reported on the following morning.

**Table 2:** Incidences of symptoms and of changes in flow-volume parameters (ΔFV) by day during a dive week.

Dive	1	2	3	4	5	later
**Wet resting O**_**2**_ **dives (WetO**_**2**_**)** n = 10						
symptoms	20%	10%	0%	10%	10%	10%
ΔFV	20%	30%	20%	30%	10%	30%
either	40%	30%	30%	40%	20%	40%
**Wet Resting air dives (WetAir)** n = 10						
symptoms	0%	10%	0%	0%	0%	0%
ΔFV	10%	10%	20%	20%	10%	0%
either	10%	10%	20%	20%	10%	0%
**Dry resting O**_**2**_ **dives (DryO**_**2**_**)** n = 14						
symptoms	17%	42%	33%	58%	50%	33%
ΔFV	17%	8%	33%	17%	0%	25%
either	25%	50%	58%	75%	50%	38%
**Wet exercise O**_**2**_ **dives (WetO**_**2**_**X**) n = 12						
symptoms	33%	50%	45%	60%	60%	60%
ΔFV	17%	17%	18%	20%	30%	20%
either	50%	67%	64%	80%	90%	80%
**Wet exercise air dives (WetAirX)** n = 14						
symptoms	7%	0%	0%	0%	0%	0%
ΔFV	7%	0%	0%	0%	7%	21%
either	7%	0%	0%	0%	7%	21%

Symptoms: inspiratory burning, cough, chest tightness or dyspnea

ΔFV: Decreases of FVC > 7.7%, FEV_1_ > 8.4%, FEF_25–75_ > 16.8% from baseline. “Either”: at least one symptom or ΔFV, sometimes both.

Note: For WetO_2_X, one diver aborted during Dive 2 and one aborted the series before Dive 4.

**Fig 1:**
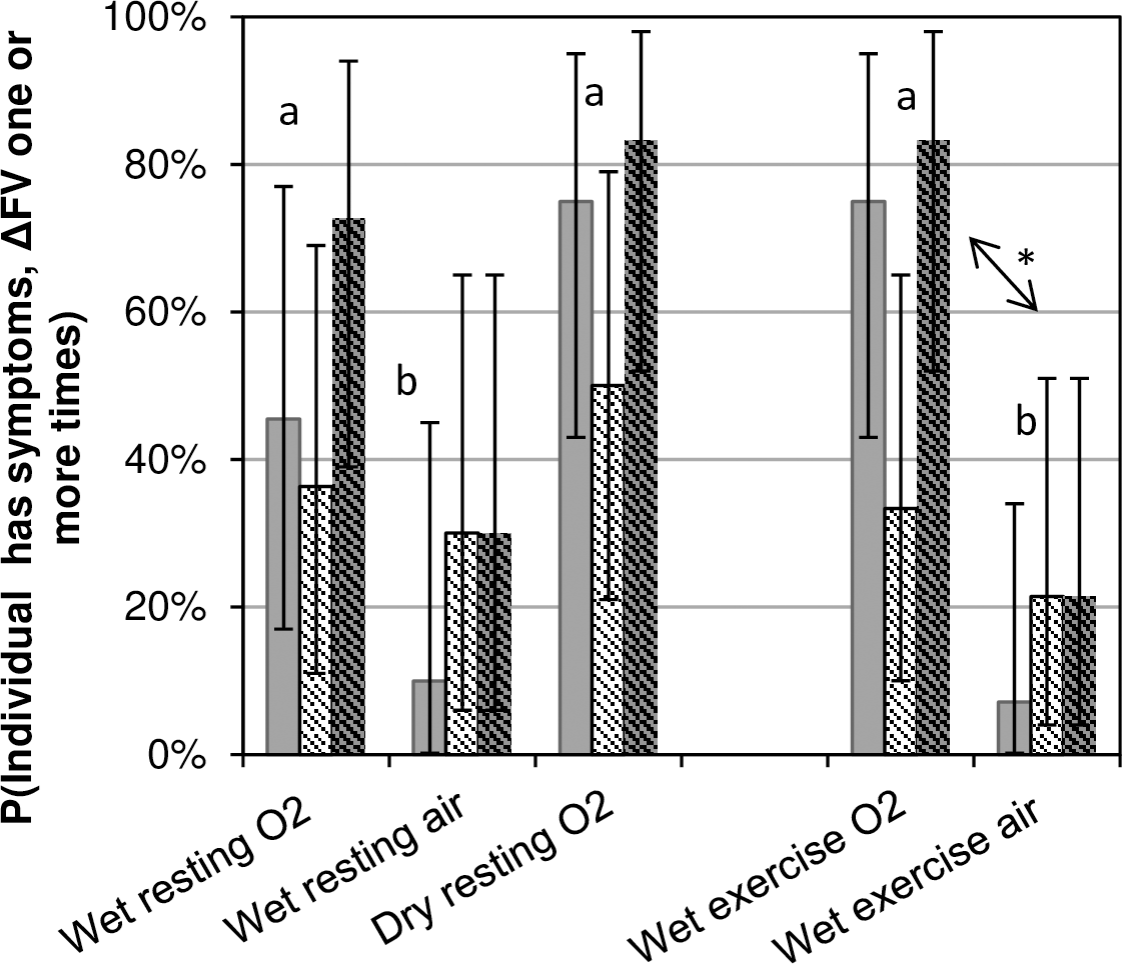
Probability of evident pulmonary oxygen toxicity on one or more days of a dive week. The incidence was calculated as the number of divers with evidence of toxicity even once during the week divided by the total number of divers. Solid grey: incidence of symptoms; hash marks: incidence of ΔFV; and grey with hash marks: symptoms and/or ΔFV, that is, any evidence of pulmonary oxygen toxicity. Error bars represent the binomial 95% confidence intervals. The statistical differences that are noted apply to the combined evidence of pulmonary oxygen toxicity. Dive conditions labeled “a” or “b” do not differ from other similarly-labeled conditions by Fisher’s Exact Test. “*” and the arrow indicate a significant pairwise difference (p<0.01). Two divers who did not complete the dive week because of symptoms (wet exercise O_2_ dives) were included in both numerator and denominator for the days when they participated.

From Table 2 one cannot differentiate between multiple individuals each affected in relation to a small number of dives and a small number of people affected across many dives. Fig 1 shows the number of individuals affected one or more times during the dive weeks. The values are the number of divers with symptoms, ΔFV, or both any time during the dive week expressed as a fraction of total number of divers for the condition. They are an estimate of the probability that any one diver will have symptoms or signs of pulmonary oxygen toxicity at any time during a dive week.

Exposures with elevated PO_2_, wet or dry, were associated with higher probabilities of symptoms and of combined symptoms and signs than were air dives. By Fisher’s Exact Test, the fraction of divers with either signs or symptoms even once during the dive week with dry resting O_2_ dives did not differ from wet resting O_2_ dives. Similarly, the number affected during wet resting O_2_ or air dive weeks did not differ from those during wet O_2_ exercise or air exercise dive weeks, respectively. The numbers during wet exercise air dive weeks were lower than those during wet exercise O_2_ dive weeks (p<0.01), and those during wet resting air dive weeks were marginally lower than those during wet resting O_2_ dive weeks (p<0.06).

**Fig 2:**
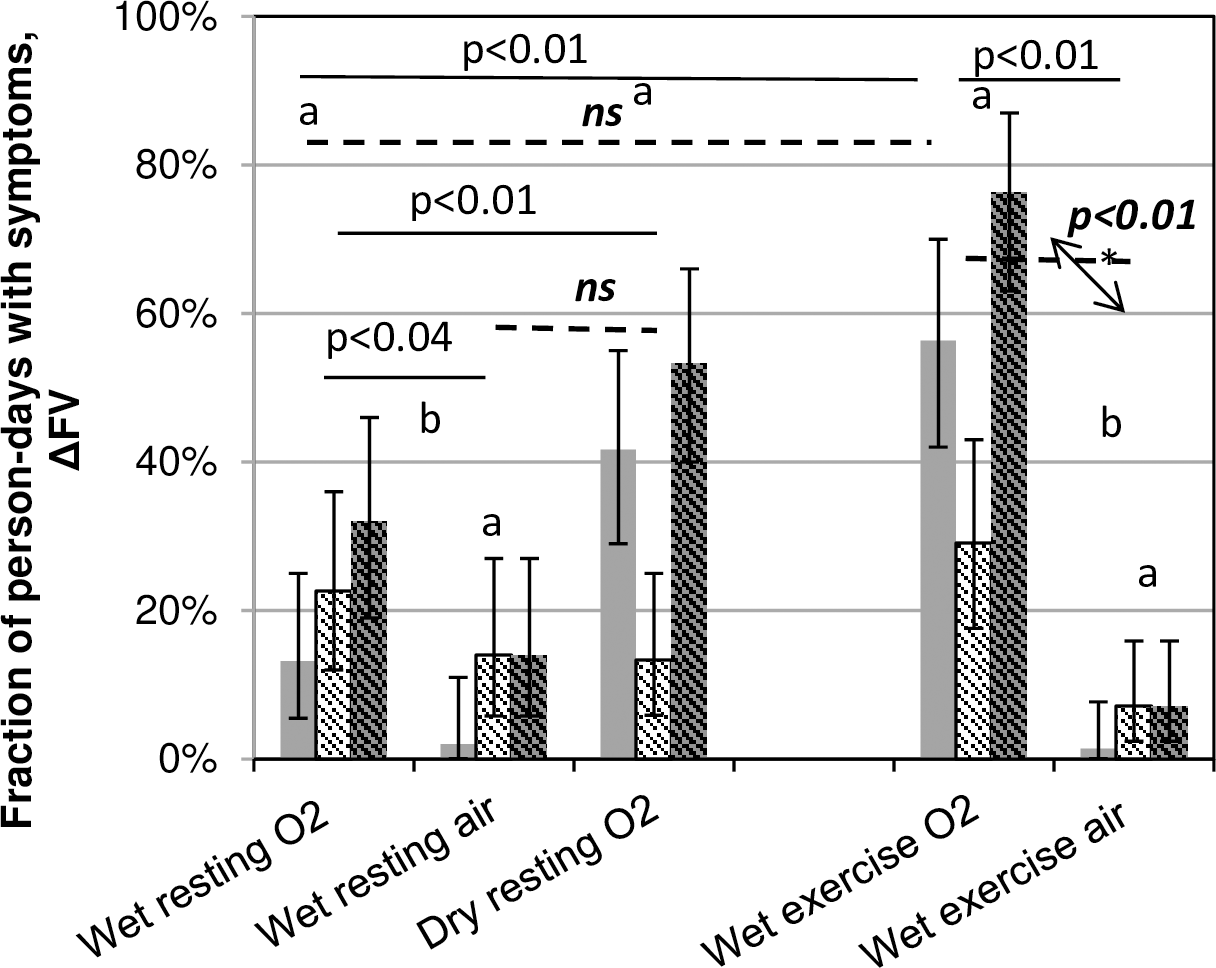
Fraction of person-days when divers showed evidence of pulmonary oxygen toxicity. Solid grey: incidence of symptoms; hash marks: incidence of ΔFV, and grey with hash marks: symptoms and/or ΔFV. Error bars represent the binomial 95% confidence intervals. Statistics in roman face represent symptoms comparisons indicated by the ends of the solid lines, and those in italics, the ΔFV comparisons indicated by the dashed lines (Fisher’s Exact Test). Dives labeled “a” do not differ from each other for either indication. Two divers who did not complete the dive week because of symptoms (wet exercise O_2_ dives) were included in both numerator and denominator for the days when they participated.

Another index, the fraction of diver-days with evidence of pulmonary oxygen toxicity (Fig 2), may give a better indication of total pulmonary insult. The number of diver-days with evidence of pulmonary oxygen toxicity was divided by the total number of diver days (e.g., 50 for ten dives for a dive week). Here also, elevated PO_2_ was associated with greater incidence of pulmonary oxygen toxicity; wet O_2_ dives showed a higher fraction of diver-days with symptoms than did wet air dives (p<0.04 at rest, p<0.01 with exercise). However, for this statistic, submergence appears protective; dry O_2_ dives had a significantly larger (p<0.01) incidence of symptoms than did wet O_2_ dives. Further, submerged exercise increased the effects of O_2_; wet resting O_2_ dives produced fewer symptoms than did wet exercise O_2_ dives (p<0.01), but wet resting and wet exercising air dives did not differ from each other.

The numbers of person-days with ΔFV showed fewer differences across conditions than did the numbers with symptoms because ΔFV was a rarer event. The number of person days with ΔFV was not different between wet resting air and dry resting oxygen, nor between resting and exercise air. Additionally, the number of person days with ΔFV did not differ between wet resting and wet exercise oxygen. However, exercise exacerbated the effects of wet O_2_ to the extent that exercise dives with O_2_ produced significantly more (p<0.01) person-days with ΔFV than did exercise dives with air.

**Fig 3:**
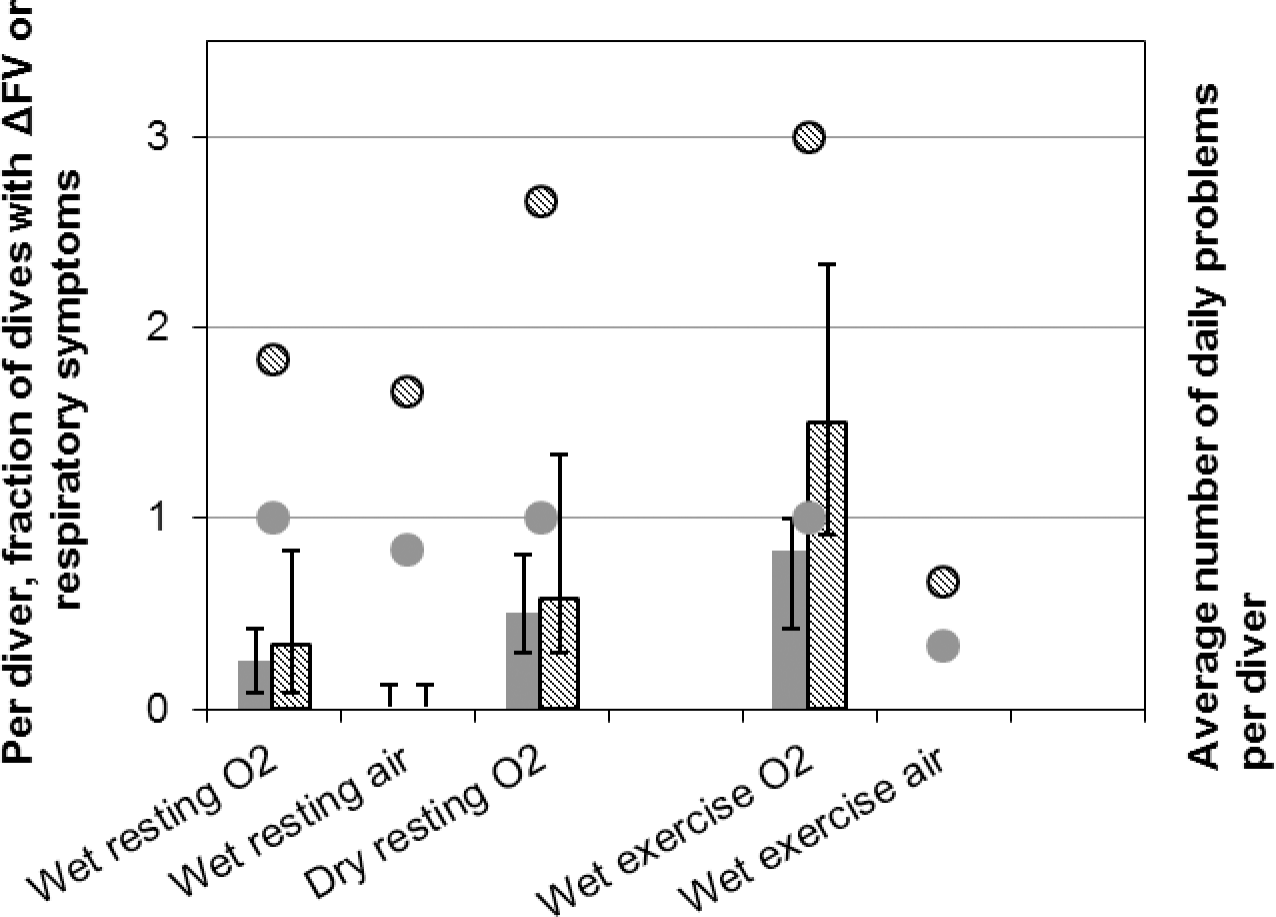
Per diver fractional signs and symptoms. Per diver fraction of dive and recovery days with ΔFV or pulmonary symptoms, and per diver average number of problems per day as functions of phase, where a “problem” is any one symptom from the list, or a deficit in FVC, FEV_1_, or FEF_25–75_. Solid gray: fraction of dive days with any signs or symptoms; hash marks: average number of problems per day. Bars: median; error bars: 1^st^ and 3^rd^ interquartile difference; round spots: maxima. The minima were zero for all conditions.

Fig 2 reflects the aggregate for all divers across the dive weeks but does not describe the variability among divers. As shown in Fig 3, the variability is quite large both for number of days with signs and symptoms, and for severity as assessed by number of separate signs and symptoms (“number of problems”) per day. The full individual tabulation appears in the Supporting Information (S1 Table, resting dives and S2 Table, dives with exercise).

Fig 3 and S1 and S2 Tables allow assessment of individual responses. The progression of the dive weeks to affect the fewest to the most divers were

1. WetAir, WetAirX: almost no one was affected, although one diver in the resting air series showed more than one change at nearly all assessments;
2. WetO_2_, DryO_2_: half of divers showed evidence of pulmonary oxygen toxicity on one quarter to one half of days and one or two divers had evidence of toxicity every day, though three divers wet and one dry had no evidence of pulmonary oxygen toxicity;
3. WetO_2_X: half of divers manifested pulmonary oxygen toxicity on nearly every assessment day, with an average of 1.5 problems per day, though one diver had no signs or symptoms on any day, and one averaged three problems per dive.

### Mean values

#### Baseline

**Fig 4:**
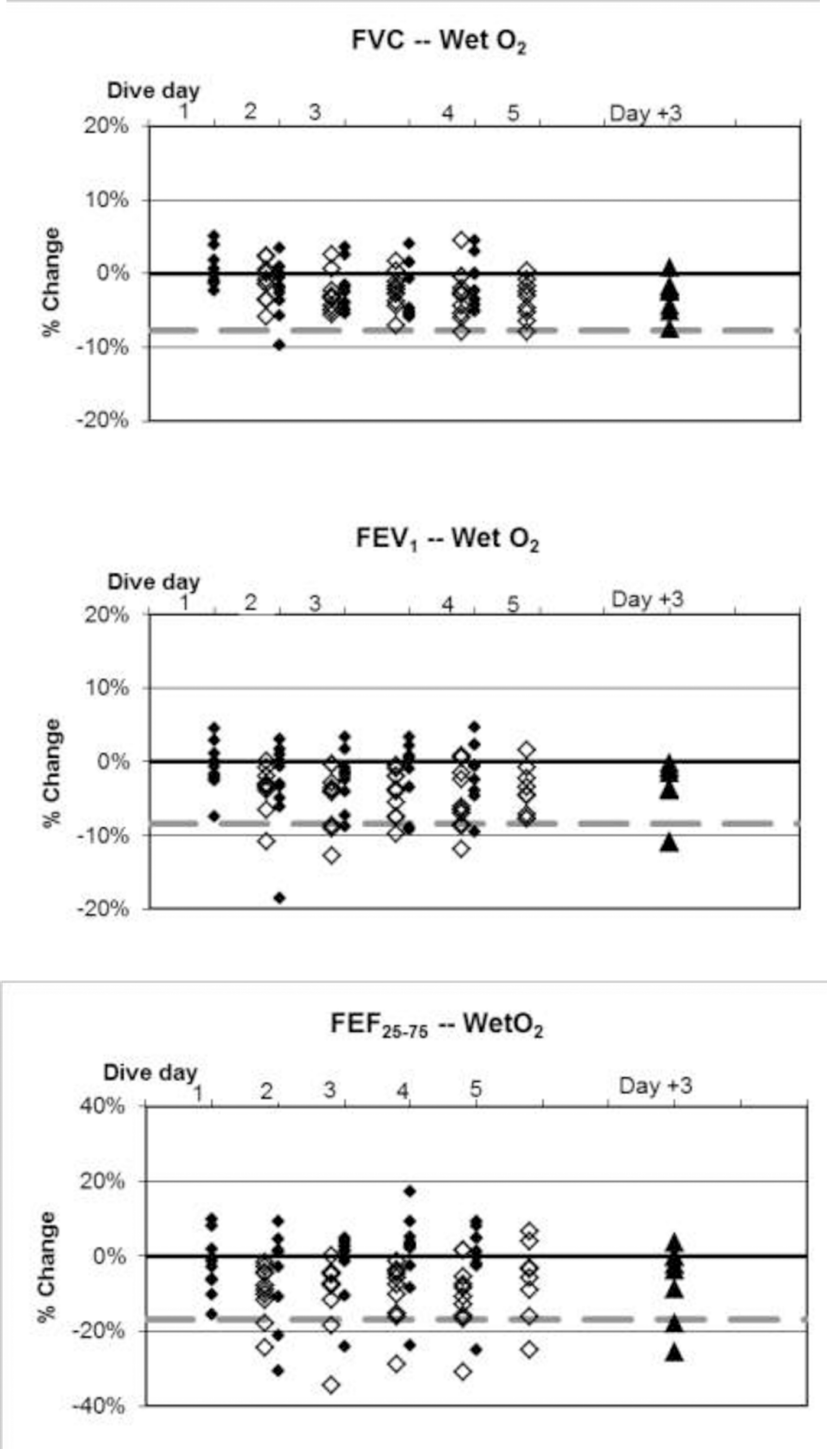
Wet resting O_2_ dives, individual differences from baseline. ♦: within 1 hour post dive; ◊: approximately 17 hours later, shown offset by 0.7 days; ▲: follow-up three days after diving ended. Dashed lines show lower limits of normal variation. The extremely low values of FEF_25–75_ are from one subject across multiple days.

**Fig 5:**
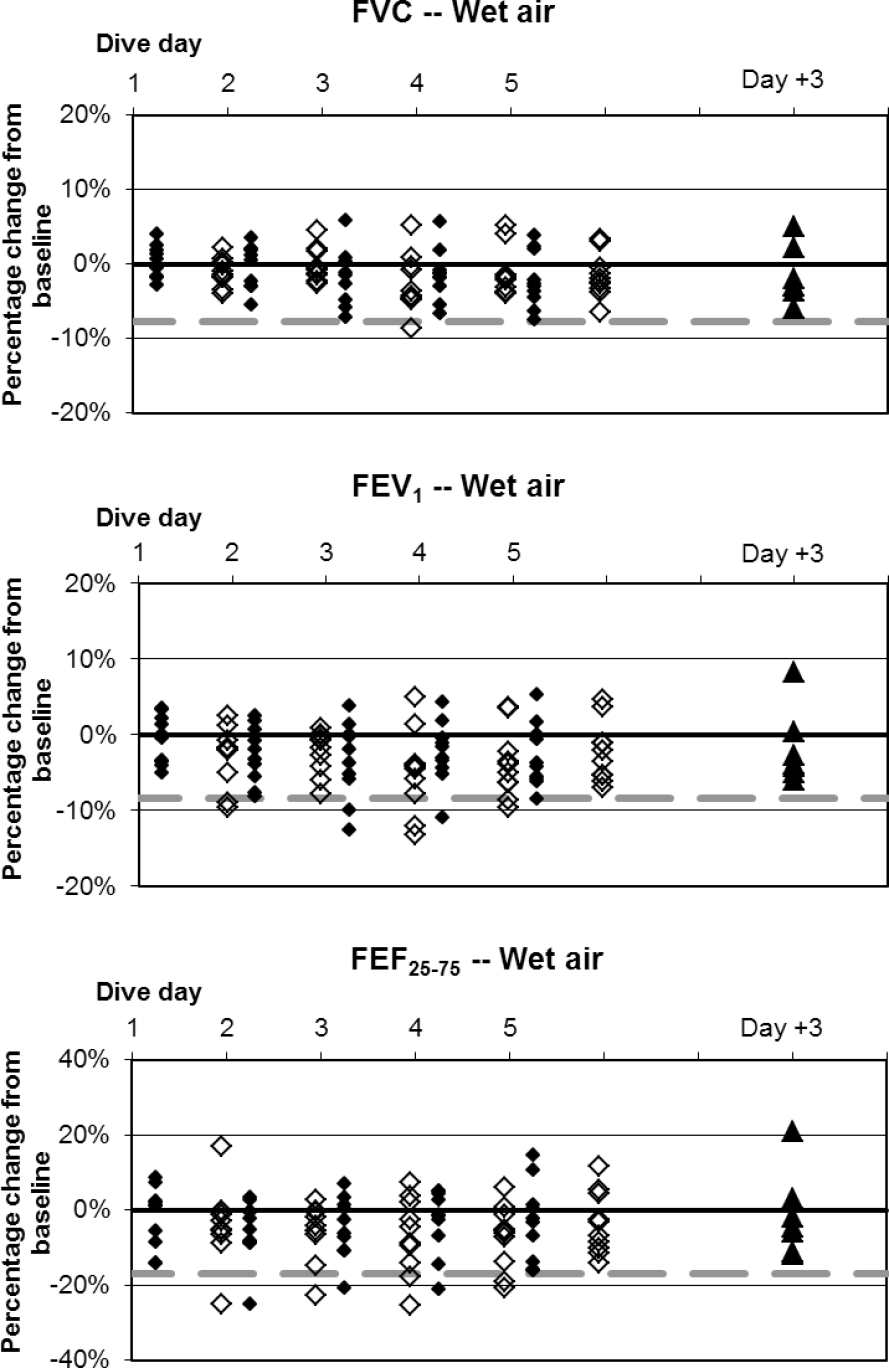
Wet resting air dives, individual differences from baseline. ♦: within 1 hour post dive; ◊: approximately 17 hours later, shown offset by 0.7 days; ▲: follow-up three days after diving ended. Dashed lines show lower limits of normal variation.

**Fig 6:**
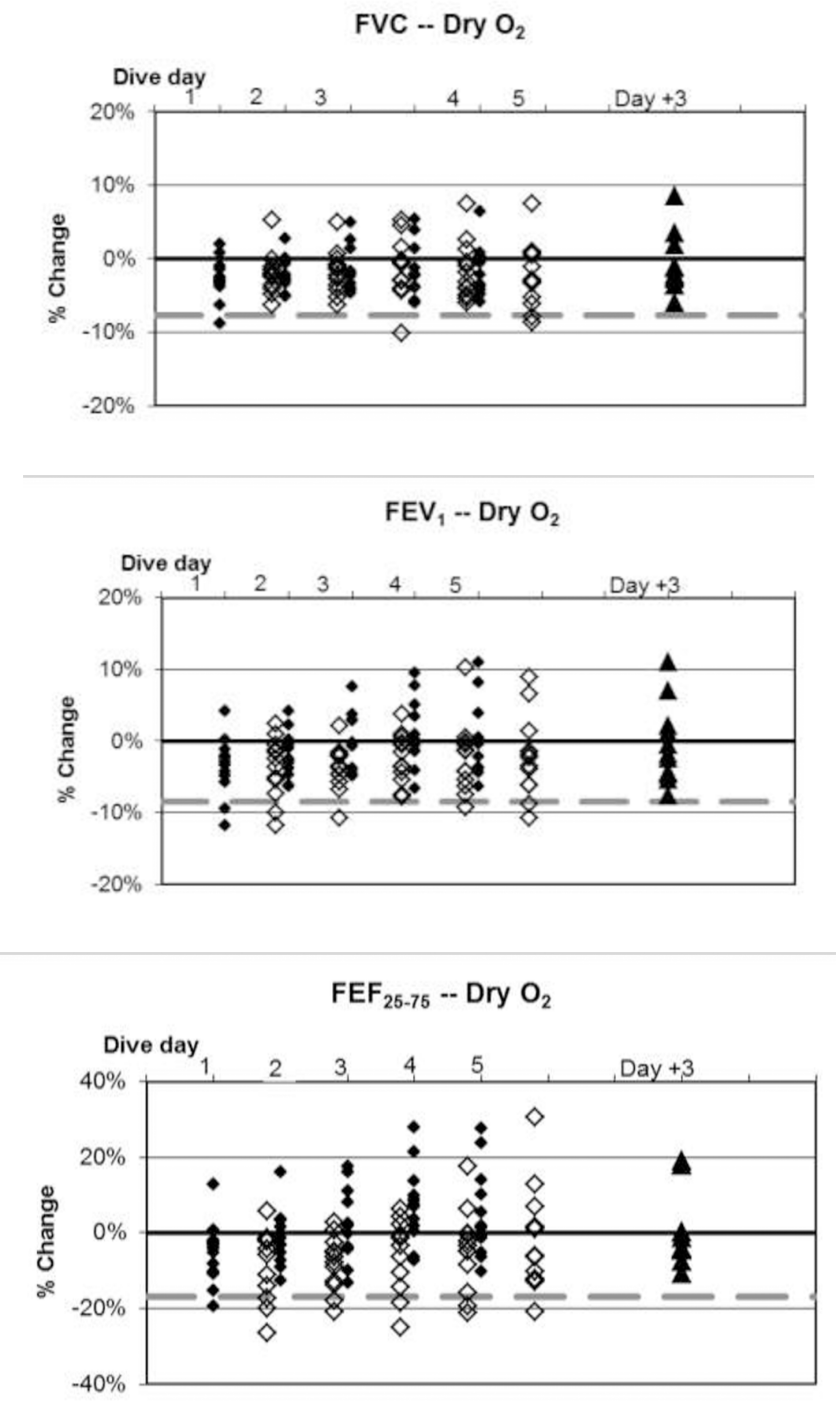
Dry resting O_2_ dives, individual differences from baseline. ♦: within 1 hour post dive; ◊: approximately 17 hours later, shown offset by 0.7 days; ▲: follow-up three days after diving ended. Dashed lines show lower limits of normal variation.

**Fig 7:**
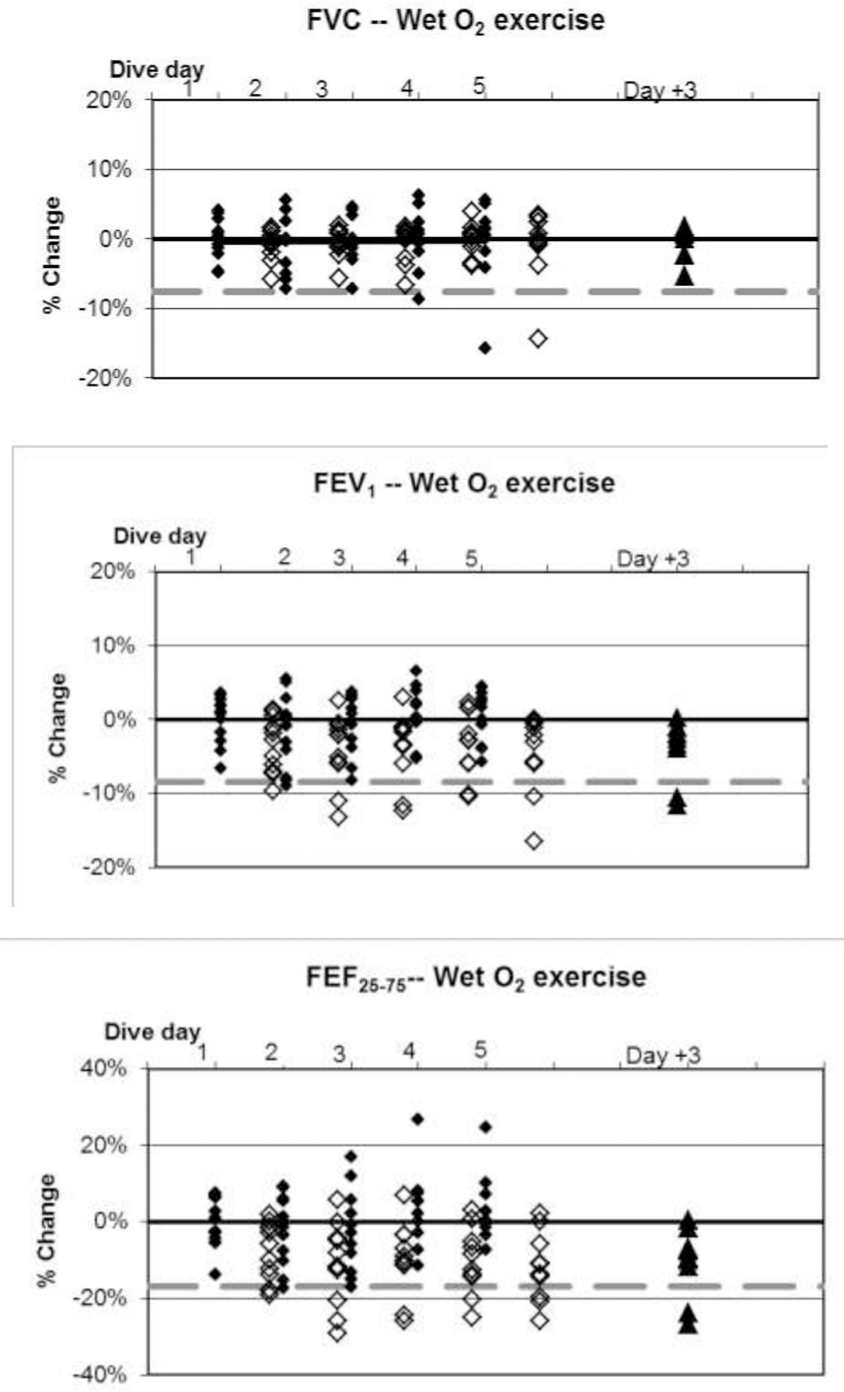
Wet O_2_ dives with exercise, individual differences from baseline. ♦: within 1 hour post dive; ◊: approximately 17 hours later, shown offset by 0.7 days; ▲: follow-up three days after diving ended. Dashed lines show lower limits of normal variation.

**Fig 8:**
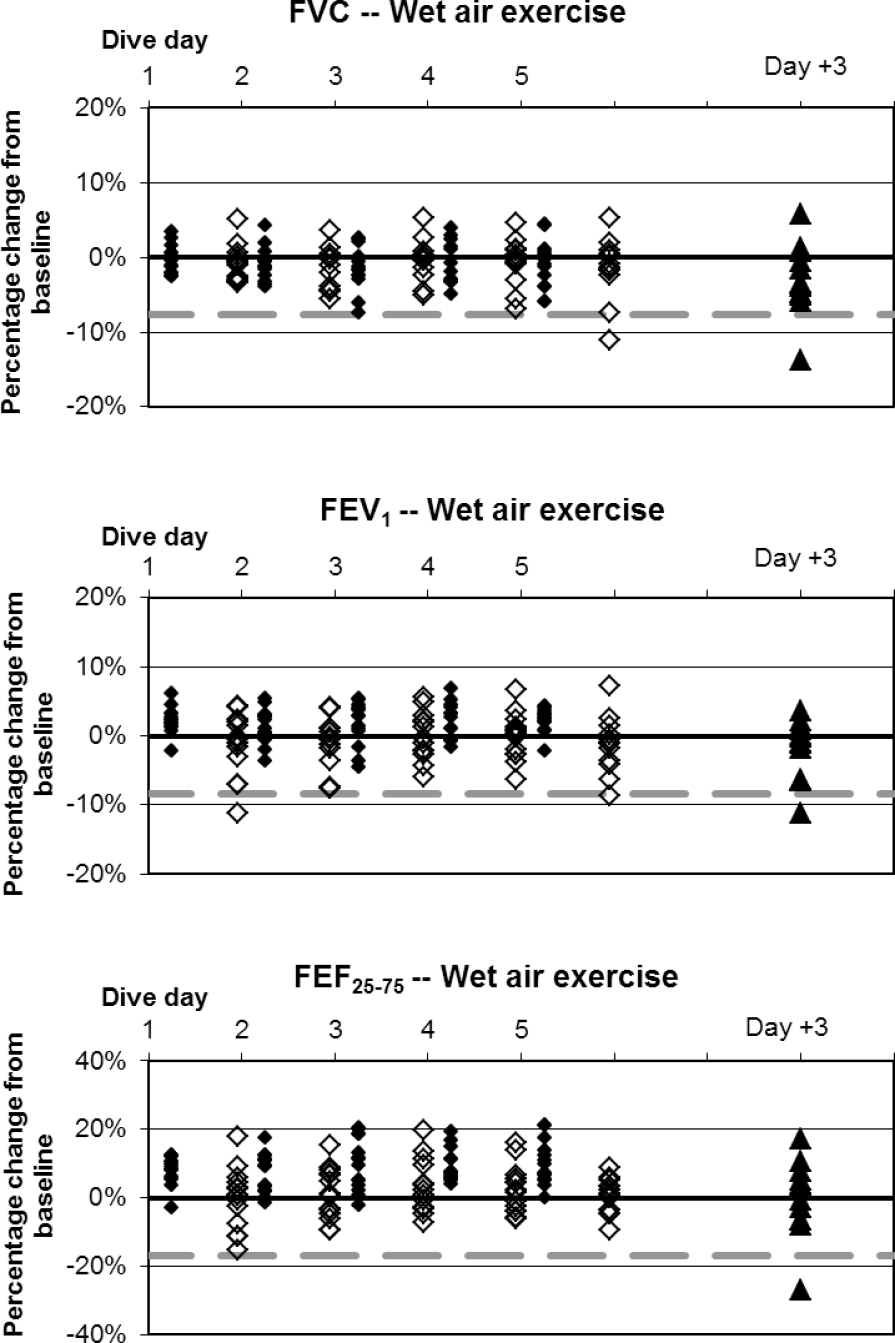
Wet air dives with exercise, individual differences from baseline. ♦: within 1 hour post dive; ◊: approximately 17 hours later, shown offset by 0.7 days; ▲: follow-up three days after diving ended. Dashed lines show lower limits of normal variation.

Baseline values are listed in Table 3. Changes in mean values of pulmonary function indices were minimal in relation to normal variation, but some statistically significant changes did occur. Fractional changes from baseline for all divers are shown in Figs 4–8.

**Table 3:** Mean values of pulmonary indices at baseline.

*Mean (standard deviation)*	FVC	FEV_1_	FEV_1_/FVC	FEF_max_	FEF_25–75_
	(L)	(L)		L/s	L/s
WetO_2_	5.88 (0.58)	4.68 (0.47)	79.5 (4.8)	11.1 (2.1)	4.40 (0.74)
WetAir	5.47 (0.58)	4.39 (0.69)	80.0 (8.2)	11.4 (1.4)	4.43 (1.64)
DryO_2_	5.78 (0.74)	4.56 (0.64)	79.0 (6.2)	11.3 (1.7)	4.16 (1.08)
WetO_2_X	5.78 (0.73)	4.66 (0.70)	80.7 (5.8)	11.6 (1.0)	4.40 (1.14)
WetAirX	6.12 (0.68)	4.61 (0.38)	75.9 (7.5)	11.2 (1.5)	3.89 (0.88)

Values are presented as means (SD).

#### Submersion effects

None of FVC, FEV_1_, or FEF_25–75_ was significantly different between WetO_2_ and DryO_2_ (both resting dives). However, resting wet and dry O_2_ exposures were not considered together because of marginal interactions of the wet-or-dry condition and dive day for some of the variables.

Within the dry dive FEV_1_ and FEF_25-75_ varied with time of day (p<0.006, p<0.003, respectively), with AM values lower than PM values. FEF_25-75_ also changed with dive day, with only AM values after Dives 1 and 2 less than baseline.

#### O_2_ effects in the water

*FVC*. There were no significant differences in FVC ascribable to breathing gas.

*FEV*_*1*_. FEV_1_ tested across all in-water dives showed no effect of breathing gas per se (no main effect), and no significant interactions of breathing gas with other variables.

*FEF*_*25–75*_. For FEF_25-75_ there was a significant effect of breathing gas (main effect p<0.008), as well as a significant interaction of breathing gas with time of day (p<0.04), and a marginal interaction with rest or exercise (p<0.06). Breathing gas had no significant effect on FEF_25-75_ PM, while FEF_25-75_ AM was significantly more depressed with O_2_ than with air (p<0.002) with no interactions with rest or exercise or dive day.

#### Exercise effects in the water

*FVC*. FVC showed only a marginal difference (p<0.07) between rest and exercise, with values slightly larger after resting dives than after exercise dives.

*FEV*_*1*_. FEV_1_ showed a strong effect of exercise (main effect p<0.005, but the exercise effect also interacted significantly with time of day (p<0.05) and with dive day (p<0.04). FEV_1_ PM was greater after exercise dives than after resting dives (p<0.002), though for neither rest nor exercise dives was any individual PM value different from baseline. However, FEV_1_ AM was only marginally different (p<0.1) between rest and exercise, with more depression from baseline after resting dives than after exercise dives. For FEV_1_ AM, rest or exercise interacted significantly (G-G p<0.003) with dive day; the exercise conditions differed significantly (exercise FEV_1_ AM was lower) only on the mornings after Dives 3 and 4. After WetAirX, FEV_1_ PM was greater than baseline after Dives 1 and 5. For all other phases and dives, it did not differ from baseline.

*FEF*_*25-75*_. There was a strong effect of exercise FEF_25–75_ (main effect <0.006), but also an interaction of exercise with time of day (p<0.03). FEF_25-75_ PM was larger after exercise dives than after resting dives (p<0.0003), not different from baseline after the resting dives but significantly elevated in the afternoon after Dives 1, 4, and 5. FEF_25-75_ AM showed only an interaction between rest and exercise and dive day; after resting dives, dive day did not significantly affect FEF_25-75_ AM, but FEF_25-75_ was depressed more after Dive 4 at rest than Dive 4 with exercise. After WetAirX, FEF_25-75_ PM was greater than baseline, and after all other phases, it did not differ from baseline for any dive.

#### Follow-up

At the follow-up measurements three days after the fifth dive, FVC and FEV_1_ were below baseline for WetO_2_, FEV_1_ and FEF_25–75_ values were below baseline for WetO_2_X, FVC was depressed for WetAirX, and a few other values remained marginally different from baseline (Table 4).

**Table 4:** Differences from baseline at three-day follow-up.

	FVC	FEV_1_	FEF_25–75_
WetO_2_	–3.0% p<0.006	–3.7% p<0.04	–6.4% (p<0.08)
WetAir	–3.6% (p<0.09)	–2.6% (p<0.07)	–3.8% n.s.
DryO_2_	–0.9% n.s.	–0.5% n.s.	–2.2% n.s.
WetO_2_X	–0.4% n.s.	–3.9% p<0.03	–10.3% p<0.01
WetAirX	–3.0% p<0.04	–2.3% (p<0.07)	+0.2% n.s.

Mean difference and statistical significance (single value t-tests) are listed.

## Discussion

The comparisons of dry oxygen to wet oxygen dives and wet oxygen to wet air dives indicate that the constellation of signs and symptoms strongly suggested pulmonary oxygen toxicity rather than an immersion phenomenon of some sort: 1) strikingly few divers had any pulmonary signs or symptoms after wet air dives, even with exercise (Fig 3); 2) incidences of signs and symptoms were high only for oxygen dives (Table 2); 3) the probability of symptoms or signs at least once during the weeks was not different among the different oxygen dives, including the dry dives, but was greater for oxygen than for air dives (Fig 1); and 3) dry oxygen dives generated more symptoms on all but the first dive day (Table 2) and more person-days with symptoms (Fig 2) than did wet oxygen dives. Thus, the hypothesis that large pressure swings in the presence of pulmonary congestion is responsible for altered pulmonary function indices and for symptoms after diving was not substantiated. Exercise exacerbated the oxygen effect without itself causing any change in signs or symptoms after air dives.

The incidence of symptoms or of ΔFV in any individual is very different from the average change in pulmonary function across a group. This study, like others reported previously [2,3], shows that average changes are minimal. Nonetheless, some individuals experiencing pulmonary oxygen toxicity have results far from the group mean (Figs 4–8). Also like the previous reports, this study shows the lack of correlation between pulmonary symptoms and pulmonary signs.

Divers generally participated in only one phase of the study. This opens the possibility that the apparent effects of the dive conditions were, in fact, individual responses; some divers may be more aware of respiratory symptoms than others. Here, the fact that divers did not participate in all phases of the study reduces the ability to separate effects of conditions from individual succeptibility. However, as differences are usually greater, not smaller, in a repeated design, we have confidence that the differences seen betweem conditions were real. Divers knew what gas they were breathing and whether or not others had reported symptoms, knowledge that could have influenced their thresholds for reporting symptoms. Even had all divers participated in all phases, group influence could not have been removed. Pulmonary function measurements, though, are objective.

### Occurrences of signs and symptoms of pulmonary oxygen toxicity

The incidences of reported symptoms following the wet resting, six-hour oxygen exposures of these dive weeks were apparently lower than those seen previously following similar dives, while the incidences of ΔFV were greater. Previous single, six-hour resting dives with PO_2_ of 130 kPa showed incidences for symptoms of 33% (95% confidence interval 20% –46%) after single dives (n = 44); 34% (18%– 54%) after two dives with an 18-hour SI (n = 29); and 44%, 50%, and 31% (n = 16) after three, four, and five resting six-hour dives with 18-hr SI, closer to those from the current dry O_2_ exposures. The concurrent incidences of ΔFV were 6% (1%– 16%) after one dive; 3% (0.1%– 18%) after two dives; and 25%, 6%, and 6% after three, four, and five dives, respectively [2]. Given the relatively small numbers of participants and the low incidences in the current dives, binomial confidence intervals are large, but the apparent difference between current and previous wet resting oxygen dive series (and between the incidence after three dives and the rest of the series) remains a puzzle.

The differences between wet and dry oxygen dives in the current series was surprising, since an earlier study showed no difference in the incidence of signs or symptoms of pulmonary oxygen toxicity between single wet and single dry six-hour dives with a PO_2_ of approximately 160 kPa, where 36% of 34 divers in both groups reported symptoms [3]. However, that study involved only one day of diving, and the current results also show no difference between the first dive of the dry and wet resting oxygen dives series. For days 2 through 5, the breathing apparatus is unlikely to have caused the difference; the slight threshold negative pressure needed to start the gas flow and the hydrostatic load of the seated posture underwater might be expected to have increased, not decreased, pulmonary injury relative to a hood with a very slight positive pressure surrounding the head. (Both devices had a slight positive pressure threshold for exhalation.) For both situations, the oxygen supply was humidified by bubbling it through water before it was delivered to the divers, although the bubblers were different (home built for the wet dives, commercial bubblers made for use with the hoods for the dry dives), and perhaps the gas delivered in the water was also humidified by water leakage into the masks. However, the difference is not that the dry dives had a higher incidence of pulmonary oxygen toxicity than that expected, but more that the wet dives had a lower incidence than had been reported earlier.

Submersion itself seems an unlikely source of protection against pulmonary oxygen toxicity. Acutely, the centralization of blood volume leads to higher pulmonary blood volume and flow and higher pulmonary capillary pressure than that in a dry environment, all factors which would promote fluid transfer into interstitial spaces through any leaky capillary walls; alveolar-capillary leaks have been detected after 17 hours of dry normobaric hyperoxia [14]. It seems reasonable to assume that a small translocated volume can generate local interstitial edema sufficient to irritate nerves without affecting overall pulmonary function parameters. However, the hypovolemia that follows from immersion [15,16,17] may have moderated the increased filtration pressures in the lungs in the wet divers despite the sustained increase in central venous pressure [18]. Hypovolemia was likely cumulative across the dive week [15], and perhaps more severe than that during the previous series of six-hour wet resting oxygen dives [2] when divers were less restricted. The sympathetic responses to hypovolemia [15,19,20] may have reduced some of the immediate post-dive symptoms; for example, terbutaline has been reported to alleviate the impaired mucociliarly clearance seen after six-hour normobaric hyperoxic exposures [21].

### Parameters of forced flow—Volume maneuvers: changes overall

None of the measured parameters changed from baseline immediately after the first dive of any of the series. However, FEV_1_ and FEF_25-75_ values about 17 hours after surfacing from the second and subsequent in-water dives were lower than those immediately post surfacing. Values immediately after surfacing from exercise dives were higher than those immediately after surfacing from resting dives. Finally, values after immersed dives with oxygen were lower than those after dives with air; in-water diving with oxygen generally depressed FEV_1_ and FEF_25–75_, and the effect persisted through the follow-up day three days after the fifth dive.

Depressed values at follow-up indicate that the values not different from or above baseline immediately post dive do not represent recovery, but rather a phenomenon of immersion or immersion with exercise. The pattern is suggestive of an inflammatory effect that begins from the morning after the first oxygen dives until several days after the last oxygen dives, but with intervening immersion (and sometimes exercise) effects that somehow increased these indices of pulmonary function. The pattern of increase without inflammation is seen for WetAirX.

Both FEV_1_ and FEF_25–75_ provide information about small airway resistance and lung elastic recoil. FEV_1_ is usually preferred in clinical practice because it is the more reproducible of the two parameters. The primary difference is that FEV_1_ spans a wider and, across subjects, a more variable range of lung volumes than does FEF_25-75_. FEV_1_ always includes information from the first 25% of the volume expired, flow dominated by resistance of the large airways. When FEV_1_/FVC is less than 75%, FEV_1_ does not include the entire middle half of expiration. Thus, when FEV_1_/FVC is low, FEV_1_ may appear normal while FEF_25–75_ shows increased small airway resistance [22]. Of these dives only the wet air exercise cohort included a large number of divers with FEV_1_/FVC ratio less than 75%, and in that cohort, FEV_1_ did not show as many changes related to diving as did FEF_25–75_.

Diving-related changes in FEV_1_ and FEF_25–75_ must be functional, not structural changes; they have rapid onset and are self-reversing. Possible sources of altered small airway resistance are changes in smooth muscle tone, degree of bronchiolar mucosal edema, airway fluid cuffing, or mucus obstruction. Lung elastic recoil could be altered reversibly by capillary engorgement or surfactant degradation.

The deficits in FEV_1_ and FEF_25–75_ on the mornings after diving but not at the initial post-dive assessment strongly suggest a post-dive inflammatory cause. Oxygen-induced injury to the airway epithelium may have caused later mucosal swelling and even smooth muscle activation. The inflammation seems to have required immersion during the oxygen exposure. This is consistent with the argument advanced above, that high PO_2_ causes capillaries to become leaky, and that the leaks become worse when a hemodynamic pressure head forces fluid through them to cause mild interstitial and perivascular edema and fluid cuffing around bronchioles and bronchial junctions. Mucus accumulation in the airways seems unlikely because cough was not a common symptom.

Restoration or increase in FEV_1_ and FEF_25–75_ immediately after both air and oxygen exposures in the water, enhanced by exercise, suggests a cause that is independent of any oxygen-induced injury. Water immersion causes lung vascular engorgement, but that has been shown to decrease lung elastic recoil at low lung volume [8], and is thus not an explanation for the increase in FEF_25–75_ shortly after dives. Further, all measurements were averages of three valid tests, and the first vital capacity maneuver can be expected to expel much of the excess blood from the pulmonary vasculature. Breakdown of pulmonary surfactant would increase lung recoil pressure, and, at least in mice, surfactant turn-over takes between 3 and 6 hours [23], which makes the time course possible, but changes in surfactant are more likely after O_2_ than air dives. Another possible cause for the early recovery of or increase in FEV_1_ and FEF_25–75_ is bronchodilation secondary to generalized increased sympathetic nervous system activity after the dives: Muscle sympathetic nervous activity increases after three days of dry immersion [19] and in response to hypovolemia [20], and six-hour water immersion induces significant hypovolemia [15]. Heart rate variability indices of sympathetic activity decrease after single six-hour resting air dives [24].

Diurnal differences may also have influenced the post-dive vs. pre-dive values. FEV_1_ is lowest during the normal sleep cycle when many of our morning measurements occurred, and highest in the early afternoon when some divers were exiting the water [25]. However, the measurement schedule was very similar for air and oxygen dives, wet or dry, while the patterns we observed were not. The time of day effect was not significant for DryO_2_. Further, the first diver of a series performed pulmonary function testing easily five hours before the last diver of a set of dives, an effect that should blur the diurnal effect [26].

### Rest vs. Exercise

The pulmonary effects differed considerably between exposures at rest and those with exercise. For air dives, apparent improvement in indices of small airway function immediately post-dive was more pronounced after exercise dives, when values after WetAirX were greater than baseline. For O_2_ dives, the overall incidence and severity (number of separate problems) of pulmonary oxygen toxicity was much greater after exercise dives than after resting dives while average changes in pulmonary function were not evident until the end of the dive week. This may be related to the further elevation by in-water exercise of pulmonary vascular pressures, already elevated by immersion at rest relative to the non-immersed state [12].

Exercising divers had a slightly lower hydrostatic respiratory load than did those resting, because they were semi-prone at a 30° head-up angle during their exercise periods rather than vertical in the water column as they were at rest. However, the exercise-induced increase in pulmonary blood volume is much greater than that for a small change in static load [11].

When divers breathed air underwater, the incidence of pulmonary signs and symptoms after diving was not increased by exercise; presumably, if the capillaries have not been damaged by hyperoxia, they don’t leak. However, when divers breathed oxygen, the incidence of pulmonary oxygen toxicity increased with exercise, possibly because increased pressures “found” more leaks. After the second or later dives the increase was more pronounced, perhaps because the inflammatory response to the first dive’s oxygen insult made the tissue more fragile. This is consistent with previous studies with four hour dives with rest or exercise, where the increase in pulmonary toxicity with exercise was not clear until the second day of diving [3].

### Conclusion

Repeated shallow six-hour dives with air as the breathing gas generate very few respiratory symptoms or reductions in pulmonary function even when the divers perform moderate aerobic work half the time; the pulmonary vasculature appears to be able to handle the immersion effects without injury. However, exposures to a PO_2_ of approximately 130 kPa cause symptoms of pulmonary oxygen toxicity and individual changes in pulmonary function parameters whether the exposure is in a dry chamber or underwater. If the underwater oxygen exposure includes moderate aerobic exercise for half the time, both incidence and individual severity of pulmonary oxygen toxicity are exacerbated. FEV_1_ and FEF_25–75_ are more affected than is FVC, and both show a pattern of late (about 17-hour) post-exposure deficit followed by improvement early after the next exposure. Evidence of increased small airway resistance remains three days after the end of in-water O_2_ dive weeks. For individuals who must exert themselves while breathing PO_2_ of 130 kPa, a schedule of 6 hours per day for more than two days is ill-advised.

To further work in this area, studies including more functional parameters should be completed. Moreover, pulmonary function should be examined in the context of more global “whole-body” oxygen toxicity that may impact diver safety and physical performance.

## Supporting information

S1 Table Incidence of pulmonary oxygen toxicity (PO_2_tox) for individual subjects at rest. A. Wet resting O_2_ (WetO_2_). B. Wet resting air (WetAir) C. Dry resting O_2_ (DryO_2_). Pulmonary oxygen toxicity in the morning after a dive is ascribed to the dive preceding it. Any reports from follow-up day post dive +1 were included with Dive 5. There are 6 possible days considered: 5 dive days, and the follow-up day, post dive +3, or day 8.
(PDF)

S2 Table Incidence of pulmonary oxygen toxicity for individual subjects who exercised under water. A. Wet O_2_ with exercise (WetO_2_X). B. Wet air with exercise (WetAirX). Pulmonary oxygen toxicity in the morning after a dive is ascribed to the dive preceding it. Any reports from follow-up day post dive +1 were included with Dive 5. There are 6 possible days considered: 5 dive days, and the follow-up day, post dive +3, or day 8.
(PDF)
