# Supplementary material for: Pulmonary effects of repeated six-hour normoxic and hyperoxic dives
Source: PLoS One. 2018 Sep 7;13(9):e0202892. doi: 10.1371/journal.pone.0202892 (PMC6128531; doi:10.1371/journal.pone.0202892)
Supplement: S1 Table — A. Wet resting O2 (WetO2). B. Wet resting air (WetAir) C. Dry resting O2 (DryO2). Pulmonary oxygen toxicity in the morning after a dive is ascribed to the dive preceding it. Any reports from follow-up day post dive +1 were included with Dive 5. There are 6 possible days considered: 5 dive days, and the follow-up day, post dive +3, or day 8. (PDF) [file pone.0202892.s001.pdf]

A. Wet resting O_2 ($WetO_2$)

Subject number	# days with PO_2tox	total # complaints	# days/ total dive days	# complaints/total dive days
**1**	0	0	0.00	0.00
**2**	1	2	0.17	0.33
**3**	5	10	0.83	1.67
**8**	0	0	0.00	0.00
**9**	1	1	0.17	0.17
**10**	3	3	0.50	0.50
**11**	6	11	1.00	1.83
**12**	1	1	0.17	0.17
**14**	2	7	0.33	1.17

B. Wet resting air (WetAir)

Subject number	# days with PO_2tox	total # complaints	# days/ total dive days	# complaints/total dive days
**1**	1	1	0.17	0.17
**2**	0	0	0.00	0.00
**3**	5	10	0.83	1.67
**4**	0	0	0.00	0.00
**5**	1	1	0.17	0.17
**6**	0	0	0.00	0.00
**7**	0	0	0.00	0.00
**8**	0	0	0.00	0.00
**10**	0	0	0.00	0.00
**11**	0	0	0.00	0.00

C. Dry resting O_2 ($DryO_2$)

Subject number	# days with PO_2tox	total # complaints	# days/ total dive days	# complaints/total dive days
**1**	3	3	0.50	0.50
**2**	4	4	0.67	0.67
**3**	3	6	0.50	1.00
**5**	1	1	0.17	0.17
**6**	2	2	0.33	0.33
**7**	3	3	0.50	0.50
**8**	1	1	0.17	0.17
**9**	0	0	0.00	0.00
**10**	6	16	1.00	2.67
**11**	5	8	1.67	2.67
**12**	4	8	0.80	1.60
**13**	6	12	1.20	2.40
