# Supplementary material for: Pulmonary effects of repeated six-hour normoxic and hyperoxic dives
Source: PLoS One. 2018 Sep 7;13(9):e0202892. doi: 10.1371/journal.pone.0202892 (PMC6128531; doi:10.1371/journal.pone.0202892)
Supplement: S2 Table — A. Wet O2 with exercise (WetO2X). B. Wet air with exercise (WetAirX). Pulmonary oxygen toxicity in the morning after a dive is ascribed to the dive preceding it. Any reports from follow-up day post dive +1 were included with Dive 5. There are 6 possible days considered: 5 dive days, and the follow-up day, post dive +3, or day 8. (PDF) [file pone.0202892.s002.pdf]

A. Wet O_2 with exercise (WetO_2X)

Subject number	# days with PO_2tox	total # complaints	# days/total dive days	# complaints/total dive days
**1**	2	5	1.00	2.50
**2**	5	9	0.83	1.50
**3**	4	8	1.33	2.67
**4**	6	9	1.00	1.50
**5**	5	9	0.83	1.50
**6**	3	7	0.50	1.17
**7**	1	1	0.17	0.17
**8**	6	18	1.00	3.00
**9**	0	0	0.00	0.00
**10**	6	14	1.00	2.33
**11**	0	0	0.00	0.00
**13**	4	14	0.67	2.33

B. Wet air with exercise (WetAirX)

**Subject number**	**# days with PO_2tox**	**total # complaints**	**# days/ total dive days**	**# complaints/total dive days**
**1**	0	0	0.00	0.00
**2**	0	0	0.00	0.00
**3**	0	0	0.00	0.00
**4**	**0**	**0**	0.00	0.00
**5**	2	3	0.33	0.50
**6**	0	0	0.00	0.00
**7**	0	0	0.00	0.00
**8**	0	0	0.00	0.00
**9**	1	2	0.17	0.33
**10**	0	0	0.00	0.00
**11**	0	0	0.00	0.00
**12**	2	4	0.33	0.67
**13**	0	0	0.00	0.00
**14**	0	0	0.00	0.00
